# „In Videobehandlungen trotz Distanz Nähe schaffen“: Wie erlebten Psychotherapeuten die Durchführung von Videobehandlungen in Zeiten von COVID-19?

**DOI:** 10.1007/s00278-021-00529-y

**Published:** 2021-08-25

**Authors:** Antje Gumz, Sulenur Kanal, Aydan Ünser, Denise Kästner, Franziska Marie Lea Beck-Hiestermann

**Affiliations:** 1 Arbeitsgruppe für Psychosomatik und Psychotherapie, Psychologische Hochschule Berlin, Am Köllnischen Park 2, 10179 Berlin, Deutschland; 2 Klinik und Poliklinik für Psychosomatische Medizin und Psychotherapie, Universitätsklinikum Hamburg‑Eppendorf, Hamburg, Deutschland

**Keywords:** COVID-19, E‑Health, „Blended care“, Kommunikationsmedien, Einstellungen, COVID-19, eHealth, Blended care, Communications media, Attitudes

## Abstract

**Hintergrund:**

Durch die im Zuge des Ausbruchs der „coronavirus disease 2019“ (COVID-19) im März 2020 erlassenen Kontaktverbote haben Psychotherapeuten deutlich mehr und die meisten von ihnen erstmalig Videobehandlungen (VB) angeboten. Bisher gibt es nur wenig Forschung dazu, wie Therapeuten die VB während der Pandemie erlebt haben, und es liegen keine Studien vor, die mögliche verfahrensspezifische Besonderheiten betrachten.

**Ziel:**

Es soll analysiert werden, welche subjektiven Erfahrungen Therapeuten unterschiedlicher Richtlinienverfahren mit der Durchführung von VB in Zeiten der COVID-19-Pandemie gemacht haben und welche Vor- und Nachteile sie erlebten.

**Methoden:**

Es handelt sich um eine „Mixed-methods“-Studie mit einer querschnittlichen Onlineerhebung. Neben quantitativen Daten wurden anhand von 7 offenen Fragen die subjektiven Erfahrungen der Therapeuten mit der Durchführung von VB erhoben und mithilfe der qualitativen Inhaltsanalyse ausgewertet. Die identifizierten Kategorien wurden einer Häufigkeitsanalyse unterzogen. Angaben von 174 ärztlichen oder psychologischen Psychotherapeuten gingen in die Auswertung ein.

**Ergebnisse:**

Besonders häufig genannte Vorteile waren die örtliche und zeitliche Flexibilität, die Kontinuität des Kontaktes in Pandemiezeiten und die Vermeidung des Infektionsrisikos. Der meistbenannte Nachteil war, dass Sinneseindrücke, Mimik, Gestik, Blickkontakt und nonverbale Kommunikation fehlen. Die meisten, aber nicht alle Patienten nahmen VB gut an. Technische Probleme erschwerten die Umsetzung.

**Schlussfolgerungen:**

Für viele Therapeuten blieb VB eine „Notlösung“, die nicht auf Dauer angelegt sei. Allerdings könnte VB über die Pandemiezeit hinaus helfen, Versorgungsprobleme (z. B. Unterversorgung auf dem Land) zu lösen. Die Ergebnisse der Studie leisten einen wichtigen Beitrag dazu, Chancen und Risiken der VB für die psychotherapeutische Versorgung abzuwägen sowie mögliche Gefahren und Schwierigkeiten im Auge zu behalten.

## Abstract (en)

**Background:**

As a result of the contact ban issued at the beginning of the coronavirus disease 2019 (COVID-19) outbreak in March 2020, psychotherapists provided significantly more video-based therapy (VBT) and most of them provided it the first time. To date, there is little research on how therapists experienced VBT during the pandemic and no studies are available that look at possible procedure-specific features.

**Objective:**

The aim was to analyze what subjective experiences therapists of different guideline procedures had with the implementation of VBT in times of the COVID 19 pandemic and what advantages and disadvantages they experienced.

**Methods:**

This was a mixed methods study with a cross-sectional online survey. In addition to quantitative data, seven open-ended questions were used to collect therapists’ subjective experiences with conducting VBT and analyzed using qualitative content analysis. The identified categories were subjected to a frequency analysis. Data from 174 medical or psychological psychotherapists were included in the analysis.

**Results:**

Particularly frequently mentioned advantages were flexibility of location and time, continuity of contact during pandemic periods and avoidance of risk of infection. The most commonly cited disadvantage was the lack of sensory input, facial expressions, gestures, eye contact, and nonverbal communication. The VBT was well-accepted by most, but not all, patients. Technical problems made the implementation difficult.

**Conclusion:**

For many therapists VBT remained a stopgap solution that was not designed to last; however, VBT could help to solve known care problems (e.g., underprovision in rural areas) beyond the pandemic period. The results of the study make an important contribution to weighing up the opportunities and risks of VBT for psychotherapeutic care and for keeping an eye on possible dangers and difficulties.

Im Zuge der durch die „coronavirus disease 2019“ (COVID-19) ausgelösten Pandemie gewannen Videobehandlungen (VB) an Bedeutung und wurden vermehrt durchgeführt. Viele Therapeuten haben ihre Bedenken oder Vorbehalte gegenüber virtuellen Therapien aufgegeben, um die Versorgung ihrer Patienten zu sichern. Dies bietet die Chance zu untersuchen, welche subjektiven Erfahrungen Therapeuten unterschiedlicher Richtlinienverfahren mit der Durchführung von VB in Zeiten der COVID-19-Pandemie gemacht haben, und welche konkreten Vor- und Nachteilen sie dabei erlebten.

## Einleitung

Die Onlinetherapie umfasst ein mannigfaltiges Spektrum an möglichen Interventionen, wie beispielsweise psychoedukative Webseiten, Selbsthilfeprogramme, Chatberatung, Schreibtherapie oder videobasierte Onlinetherapiesitzungen. Der vorliegende Beitrag beschäftigt sich ausschließlich mit den videobasierten Onlinetherapiesitzungen (Videobehandlungen, Videosprechstunden [VB]).

Während der COVID-19-Pandemie wurde der VB eine für den Versorgungalltag bisher nicht dagewesene Relevanz zuteil. Die im Zuge der Pandemie im März 2020 erlassenen Kontaktverbote und Appelle zur sozialen Distanzierung führten vielfach zu einem Wechsel von „Face-to-face“-Therapie zur VB (Liu et al. 2021; Eichenberg 2021). In kürzester Zeit mussten sich Psychotherapeuten und Patienten auf neue Bedingungen einstellen. Der VB kam und kommt in der aktuellen Pandemiesituation unvorhergesehen eine zunehmend größere Bedeutung zu (Wind et al. 2020).

Psychotherapie via digitalem Format ist keine neue Entdeckung (Wittson et al. 1961). Da der Nutzen von VB allerdings kontrovers diskutiert wird, gab es präpandemisch eine Beschränkung der Abrechnungsmöglichkeit auf anteilig maximal 20 %. Dies limitierte die Verbreitung (BPtK 2020), obwohl die Wirksamkeit prinzipiell gut belegt ist (Andersson et al. 2019). In der Pandemie wurde das Limit aufgehoben, und es kam zu einem sprunghaften Anstieg der Nutzung während des ersten Lockdowns von März bis Mai 2020 auf durchschnittlich 43 % (Beck-Hiestermann et al. 2021). Dabei traten Therapeuten der VB mit verschiedenen Einstellungen gegenüber und bewerteten diese i. Allg. als „akzeptabel“ bis „gleichwertig“ (Connolly et al. 2020). Ein systematisches Review zeigte, dass Vorerfahrung mit VB sowie die berufliche Erfahrung positiv beeinflussten, mit welcher Einstellung die Therapeuten an die Umstellung herangegangen sind (Békés und Aafjes-van Doorn 2020). Trotz dieser Erkenntnisse ist es der Einmaligkeit der Situation immanent, dass es bisher wenig Forschung zum subjektiven Erleben und den Erfahrungen der Therapeuten mit VB während der Pandemie gibt. Vor dem Hintergrund der wachsenden Bedeutung von VB im Allgemeinen (ländliche Unterversorgung, Erreichbarkeit vulnerabler Gruppen) und in der Pandemie im Besonderen ist es sinnvoll, Therapeuten zu diesen Punkten zu befragen. Die Erfahrungen von Klinikern können einen wichtigen Beitrag dazu leisten, die Unterschiede zwischen Face-to-face-Therapie und VB genauer zu verstehen. Die Telemedizin erscheint in einer Zeit mit Sparzwang im Gesundheitswesen schnell als Allheilmittel, da die Gesundheitsversorgung häufig kostengünstiger und schneller verfügbar wird. Dies birgt gleichzeitig die Gefahr der Entpersonalisierung im Zwischenmenschlichen. Die Pandemie führte dazu, dass viele Psychotherapeuten damit konfrontiert wurden, VB zu erproben. Dies ermöglicht einen erfahrungsbasierten vorurteilsfreieren Blick auf die Chancen und Risiken der VB.

Unseres Wissens existiert bisher lediglich eine niederländische Studie, die sich qualitativ mit den Erfahrungen der Therapeuten mit VB während der Pandemie (Feijt et al. 2020) auseinandersetzt. Im Ergebnis zeigte sich, dass technologische und Bedienungsprobleme eine Herausforderung darstellten. Weiterhin wurde festgestellt, dass sich VB nicht für alle Störungsbilder eignet, und teilweise wurden Schwierigkeiten beschrieben, online eine therapeutische Beziehung aufzubauen. Dennoch überwogen die positiven Aspekte: Flexibilität, Niedrigschwelligkeit und Zeitersparnis aufgrund fehlender Arbeitswege (Feijt et al. 2020). In Deutschland existiert eine „Blitzumfrage“ der Deutschen Psychotherapeutenvereinigung (DPtV 2020), die zeigen konnte, dass deutsche Therapeuten offen gegenüber VB waren, allerdings zog die Mehrheit den persönlichen Kontakt aus therapeutischen Gründen vor. Nicht berücksichtigt wurden potenzielle Unterschiede zwischen den Richtlinienverfahren.

Erfahrungen von Klinikern können einen wichtigen Beitrag dazu leisten, Chancen und Risiken der VB für die psychotherapeutische Versorgung unterschiedlicher Patientengruppen jenseits von Vorurteilen abzuwägen und mögliche Gefahren und Schwierigkeiten im Auge zu behalten. Auch angesichts der großen Relevanz der Überzeugtheit der Therapeuten vom angewandten Verfahren („allegiance“; Wampold et al. 2018) ist die Perspektive der Versorger essenziell: Wären Therapeuten von dem Format nicht überzeugt, wäre zu erwarten, dass die Behandlungen weniger effektiv sind. Vor diesem Hintergrund soll in vorliegender Studie analysiert werden, welche subjektiven Erfahrungen Therapeuten unterschiedlicher Richtlinienverfahren mit der Durchführung von VB in Zeiten der COVID-19-Pandemie gemacht haben, und welche Vor- und Nachteile sie erlebten.

## Methode

### Studiendesign und Rekrutierung

Es handelt sich um eine querschnittliche „Mixed-methods“-Studie (qualitative Kategorienbildung mit anschließender quantitativer Häufigkeitsanalyse). Die Rekrutierung erfolgte vom 01.12.2020 bis 31.12.2020. Die Daten wurden anonym online erhoben (Umfrage-Software EFS Survey; Questback GmbH 2015, Berlin, Deutschland). Es wurden alle Ausbildungsinstitute in Deutschland sowie 5965 niedergelassene, in Therapeutensuchmaschinen gelistete Psychotherapeuten per E‑Mail kontaktiert. Des Weiteren wurden soziale Netzwerke zur Rekrutierung genutzt. Zur Verbreitung im Sinne eines „Snowball-Effektes“ befand sich am Ende der Umfrage ein Text zur Weiterleitung an Kollegen.

Befragt wurden ärztliche oder psychologische Psychotherapeuten aller Richtlinienverfahren (systemische Therapie, ST; analytische Psychotherapie, AP; tiefenpsychologisch fundierte Psychotherapie, TP; Verhaltenstherapie, VT). Ausgeschlossen wurden andere Behandler, beispielsweise Heilpraktiker oder Coaches. Einschlusskriterien waren: therapeutische Erfahrung (approbiert oder in fortgeschrittener Ausbildung, Behandlungsphase), die Durchführung von mindestens einer onlinetherapeutischen Sitzung während des ersten Lockdowns von März bis Mai 2020 (jeweils erhoben durch Selbsteinschätzung).

Die Erhebung beinhaltete einen quantitativen Teil mit u. a. soziodemografischen Angaben und Daten zu therapeutischer Tätigkeit und Ausbildung. Zudem wurden 7 offene Fragen in einem iterativen Diskussionsprozess im Forschungsteam entwickelt. Orientiert an Empfehlungen für Experteninterviews (Helfferich 2011) wurden, ausgehend von der übergeordneten Forschungsfrage, wie Therapeuten die VB während der Pandemie erlebten, 7 Subdimensionen erarbeitet. Für diese Subdimensionen wurden spezifische Fragen entwickelt, die direkt auf das interessierende Forschungsthema abzielten (statt offener, erzählauffordernder Fragen, die stärker bei Befragungen von Laien zum Einsatz kommen). Die spezifischen Fragen wurden abschließend Klinikern, die in die Fragebogenentwicklung nicht involviert waren, zu einer Pretestung vorgelegt (4 approbierte Psychotherapeuten/Psychotherapeutinnen, 8 Psychotherapeuten/Psychotherapeutinnen in Ausbildung, Prüfen von Verständlichkeit der Fragen, Varianz in den Antworten, Aussagekraft und Vollständigkeit). Die 7 in Freitextfeldern erhobenen Fragen lauteten:

1. Welche Vorteile hat VB (z. B. für Sie persönlich, für die Therapie oder für die Patienten)?
2. Welche Nachteile hat VB (z. B. für Sie persönlich, für die Therapie oder für die Patienten)?
3. Was hat Ihnen bei der Umstellung auf VB geholfen?
4. Welche Einflüsse hatte der Wechsel zu VB auf die Therapieverläufe?
5. Wie waren die Reaktionen Ihrer Patienten beim Wechsel zur VB?
6. Gab es Patienten, bei denen Sie den Wechsel zu VB als einfacher/unproblematischer erlebt haben? Falls ja, bitte erläutern Sie.
7. Haben sich Ihre Ansichten zum Thema VB seit Beginn der COVID-19-Pandemie verändert? Falls ja, bitte geben Sie hier an, inwiefern sich diese geändert haben.

### Datenanalyse

Die Daten wurden mithilfe der qualitativen Inhaltsanalyse ausgewertet (Mayring 2015). Die folgende Darstellung des Kodierablaufs folgt den Standards for Reporting Qualitative Research (O’Brien et al. 2014): Zunächst wurden die Kategorien von einem Primärteam (2 Studentinnen im Master Psychologie; S.K., A.Ü.) induktiv gebildet; einzelne Aussagen wurden paraphrasiert sowie zu Ober- und Subkategorien zusammengefasst. Das Primärteam war hinsichtlich der sonstigen quantitativen Daten und Angaben zu den Therapeuten verblindet. Das Kategoriensystem wurde im zweiten Schritt durch eine Auditorin (Professorin für Psychosomatik und Psychotherapie, analytische, tiefenpsychologisch fundierte und systemische Psychotherapeutin, A.G.) überarbeitet, d. h., alle Einzelaussagen wurden im Hinblick auf ihre Zuordnung zu Sub- und Oberkategorien geprüft, Sub- und Oberkategorien wurden im Hinblick auf Vollständigkeit und Bezeichnung geprüft. Im dritten Schritt wurden die Kategorien und deren Zuordnung auf dieselbe Weise von einem Sekundärteam geprüft, bestehend aus einer promovierten Psychologin in Ausbildung zur tiefenpsychologisch fundierten Psychotherapeutin (D.K.) sowie einer Psychologin und Doktorandin im postgradualen Masterstudium TP (L.B.), Diskrepanzen wurden markiert; hieran anschließend fand im Gesamtteam eine Konsensfindung statt.

Zuverlässigkeit und intersubjektive Nachvollziehbarkeit wurden zudem durch die Vorstellung und Diskussion der Studienergebnisse in einem Forschungskolloquium geprüft. Persönliche Vorannahmen und Erwartungen waren: S.K. erwartete, dass Therapeuten mit Vorerfahrungen VB in der Pandemiezeit auch besser umsetzen konnten, und dass AP- und TP- mehr Nachteile benennen als VT-Therapeuten. A.Ü. erwartete, dass VB eine Bereicherung darstellt, jedoch die Face-to-face-Therapie nur temporär ersetzen wird. Darüber hinaus vermutete sie, dass bei VTlern der Übergang zur VB aufgrund ihrer Therapiestruktur und Affinität zu Onlinekonzepten reibungsloser verlaufen wird. A.G. erwartete stärkere Vorbehalte und erlebte Nachteile der VB unter psychodynamischen Kollegen, v. a. in Bezug auf den Umgang mit Übertragung und Gegenübertragung in der therapeutischen Beziehung, nonverbale Prozesse und Resonanz. D.K. hatte die Annahme, dass bei einer zuvor geringen Nutzung von VB der Wechsel Umstellungskosten verursachte und laufende Therapieprozesse verschiedenartig beeinflusste. Eine kritischere Haltung erwartete sie mit Hinblick auf neue Patienten, im Bereich Kinder- und Jugendlichenpsychotherapie (KJP) sowie bei älteren und analytisch arbeitenden Therapeuten. L.B. erwartete, dass ältere Therapeuten tendenziell mehr Nachteile bei der VB sehen als jüngere, und dass es in Abhängigkeit vom Richtlinienverfahren eine deutliche Präferenz (VT) bzw. Ablehnung von VB (AP) gibt.

Mit den identifizierten Kategorien wurde eine Häufigkeitsanalyse durchgeführt, bei der die Nennungshäufigkeit allgemein sowie in Abhängigkeit vom Richtlinienverfahren betrachtet wurde.

## Ergebnisse

### Stichprobenbeschreibung

Die 174 befragten Psychotherapeuten (Alter: 28 bis 78 Jahre, M = 44,73 Jahre, SD ± 12,79 Jahre; 81,6 % Frauen, 17,4 % Männer) verteilen sich wie folgt auf die Verfahren: *n* = 10 (5,8 %) ST, *n* = 24 (14 %) AP, *n* = 59 (34,5 %) TP, *n* = 78 (45,6 %) VT; drei Teilnehmende (1,7 %) machten keine Angabe. Soziodemografische Daten und Vorerfahrungen mit VB sind Tab. 1 zu entnehmen.

**Table 1:** 

	***M***	***±*** ***SD***
**Alter (Jahre)**	44,73	**±** 12,79
	***n***	**%**
**Geschlecht**		
Männlich	30	17,4
Weiblich	142	81,6
Ohne Angabe	2	1,2
**Familienstand**		
Ledig	59	33,9
Verheiratet	91	52,3
Getrennt/geschieden	18	10,3
Sonstiges	6	3,5
**Studium**		
Psychologie	138	79,3
Medizin	15	8,6
Sonstiges	21	12,1
**Entscheidung für VB**		
Eigene Entscheidung, ob VB oder nicht	154	88,5
Arbeitgeber lehnte VB ganz oder teilweise ab	11	6,3
Arbeitgeber gab VB ganz oder teilweise vor	9	5,1
**Vorerfahrungen mit VB**		
„Ja“	41	23,6
„Nein“	132	75,9
Ohne Angabe	1	0,6

*VB* Videobehandlung

### Ergebnisse der qualitativen Analyse

Von 174 Probanden beantworteten 149 (87,1 %) die Freitextfelder. Die Response-Rate verteilte sich wie folgt auf die Richtlinienverfahren: 100 % der ST, 87,5 % der AP, 87,2 % der VT und 87,5 % der TP. So kamen 1392 schriftliche Einzelaussagen zusammen, die thematisch in 88 Sub- und 9 Oberkategorien geordnet wurden.

**Figure 1:**
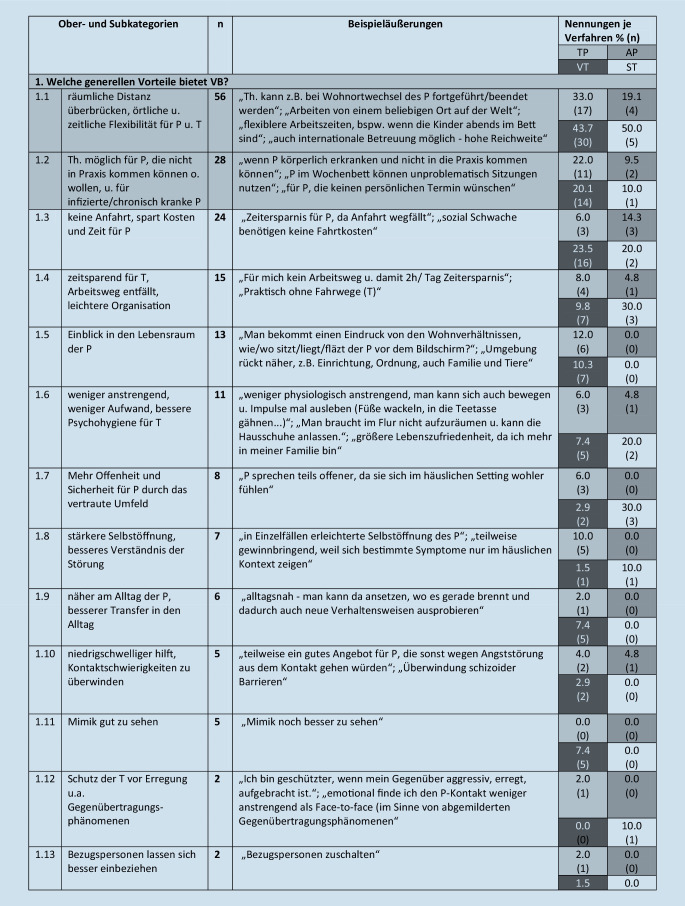

Diese werden im folgenden Text beschrieben, wenn sie von mehr als 5 Teilnehmern benannt wurden. Eine Übersicht über alle Kategorien (einschließlich von *n* ≤ 5 Teilnehmenden benannte), illustrative Beispieläußerungen sowie Angaben zur Nennungshäufigkeit in Abhängigkeit vom Richtlinienverfahren gibt Abb. 1.

**Figure 2:**
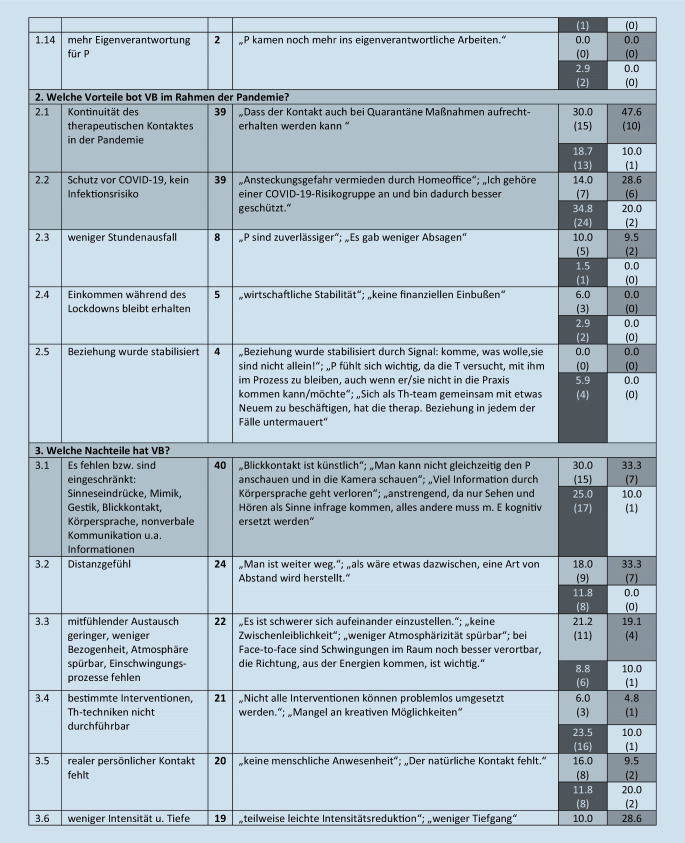

**Figure 3:**
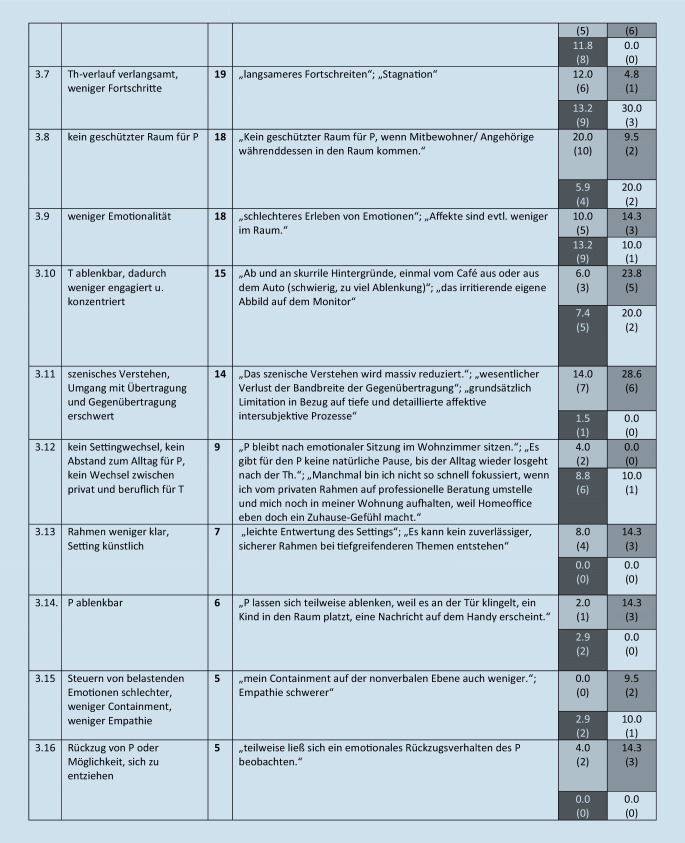

**Figure 4:**
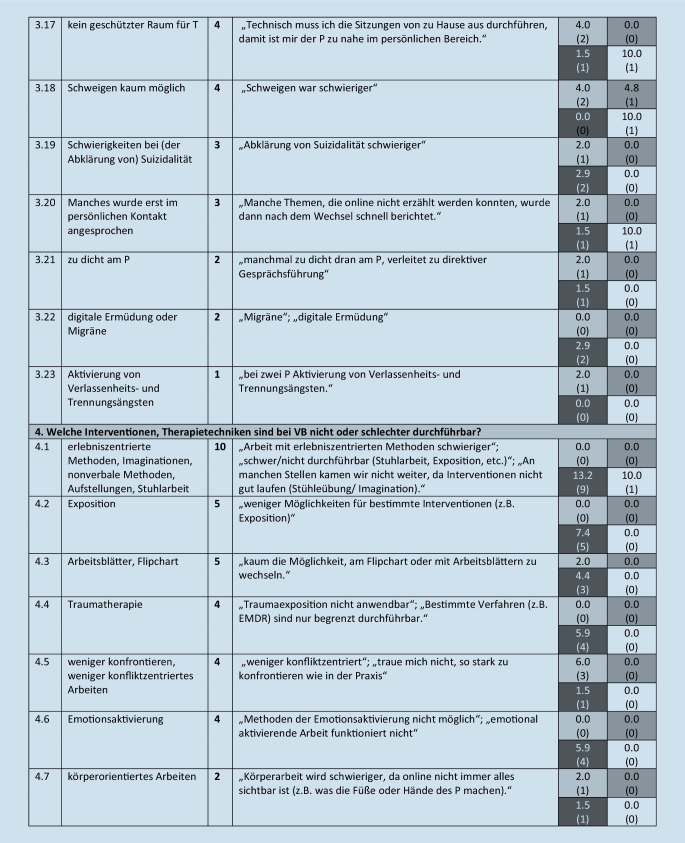

**Figure 5:**
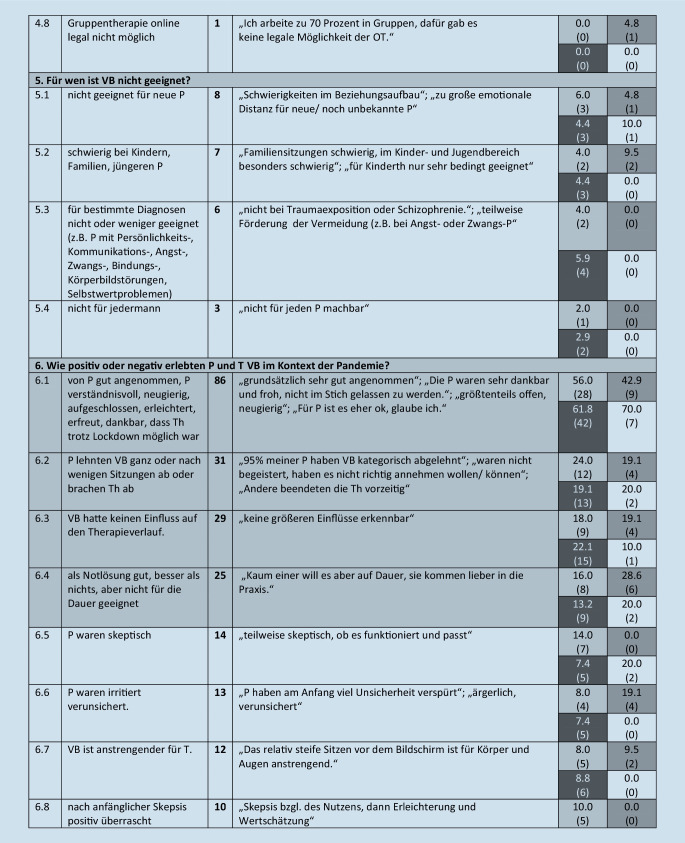

**Figure 6:**
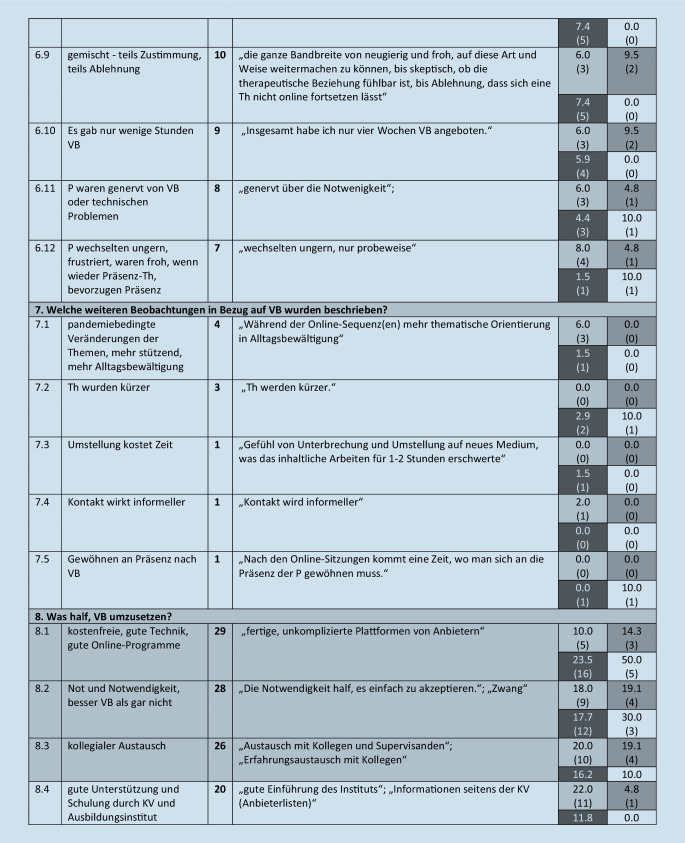

**Figure 7:**
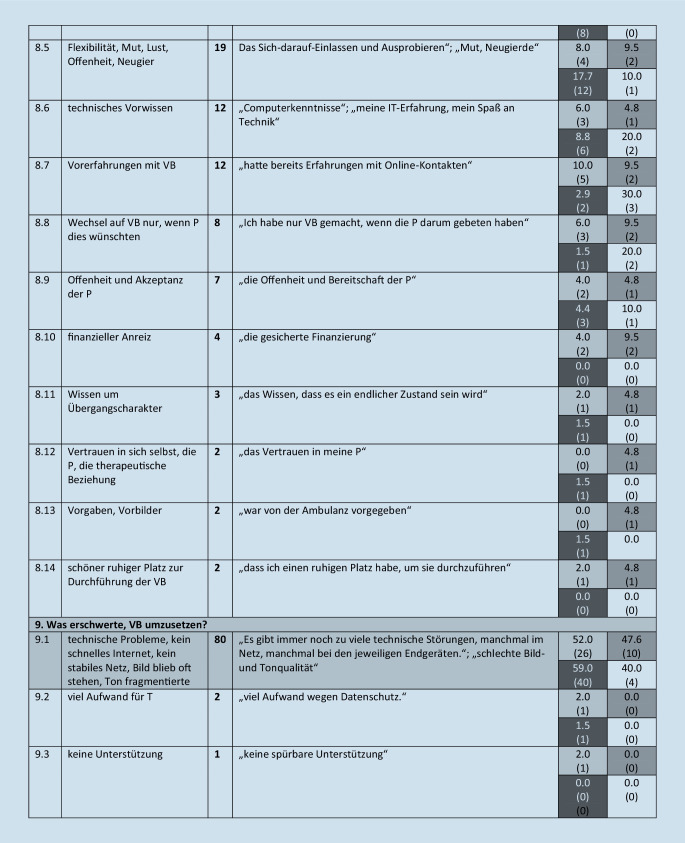

#### 1. Oberkategorie: Welche generellen Vorteile bietet VB?

Die Oberkategorie umfasst 14 Subkategorien. Am häufigsten benannt (von *n* = 56, etwa 32 % der Therapeuten) wurde der Vorteil, dass VB räumliche Distanz überbrücken kann sowie örtliche und zeitliche Flexibilität für Patienten und Therapeuten ermöglicht (Kategorie 1.1). Etwa 16 % der Therapeuten (*n* = 28) beschrieben Vorteile, die sich darauf beziehen, dass VB eine Therapiemöglichkeit für Patienten bietet, die nicht in die Praxis kommen können oder wollen, z. B. für infizierte oder chronisch kranke Patienten (1.2). Etwa 14 % (*n* = 24) erwähnten, dass VB Anfahrtswege, Kosten und Zeit für Patienten ersparen kann (1.3), 9 % (*n* = 15) benannten auch die Zeitersparnis für Therapeuten, den wegfallenden Arbeitsweg und leichtere Organisation (1.4). Von den Teilnehmenden berichteten 7 % (*n* = 13), dass VB einen Einblick in den Lebensraum der Patienten ermöglicht (1.5). Weitere erlebte Vorteile: Eine VB ist weniger anstrengend, weniger aufwendig für Therapeuten und ermöglicht eine bessere Psychohygiene (1.6; 6 %, *n* = 11), bietet mehr Offenheit und Sicherheit für die Patienten durch das vertraute Umfeld (1.7; 5 %, *n* = 8), eine erlebte stärkere Selbstöffnung der Patienten und damit ein besseres Verständnis der Störung (1.8; 4 %, *n* = 7). Eine VB ist näher am Alltag der Patienten, oder der Transfer in den Alltag gelingt besser (1.9; 3 %, *n* = 6).

#### 2. Oberkategorie: Welche Vorteile bot VB im Rahmen der Pandemie?

Diese umfasst 5 Subkategorien. Jeweils etwa 22 % der Therapeuten (*n* = 39) beschrieben den Vorteil, dass die Kontinuität des therapeutischen Kontakts zu Zeiten der Pandemie aufrechterhalten werden konnte, bzw. dass es keine Therapieunterbrechung gab (2.1) sowie dass die VB vor COVID-19 bzw. dem Infektionsrisiko schützte (2.2). Fünf Prozent formulierten, dass es durch die Möglichkeit der VB weniger Stundenausfall gab (*n* = 8, 2.3).

#### 3. Oberkategorie: Welche Nachteile hat VB?

Diese umfasst 23 Subkategorien. Ein knappes Viertel der teilnehmenden Therapeuten (23 %, *n* = 40) beschrieb das Fehlen von Sinneseindrücken, Mimik, Gestik, Blickkontakt, Körpersprache, nonverbaler Kommunikation und anderer Informationen (Kategorie 3.1). Ein Distanzgefühl beschrieben 14 % (*n* = 24; 3.2), 13 % (*n* = 22) schrieben, dass der mitfühlende Austausch geringer ist, weniger Atmosphäre spürbar ist, und dass Einschwingungsprozesse fehlen (3.3), 12 % (*n* = 21) schrieben, dass bestimmte Interventionen oder Therapietechniken nicht durchführbar sind (3.4), jeweils etwa 11 % (*n* = 20 bzw. *n* = 19) empfinden, dass der reale persönliche Kontakt und die Präsenz fehlen (3.5), erleben weniger Intensität und Tiefe (3.6) und benannten den Nachteil, dass die Patienten bei VB keinen geschützten Raum haben (3.8), und dass der Therapieverlauf verlangsamt ist und weniger Fortschritte erzielt werden (3.7), etwa 10 % (*n* = 18) erleben bei VB weniger Emotionalität (3.9).

Etwa 9 % (*n* = 15) schrieben, bei VB ablenkbarer zu sein, dadurch weniger engagiert, weniger konzentriert zu arbeiten (3.10), 8 % (*n* = 14) erwähnten, dass das szenische Verstehen oder der Umgang mit Übertragung und Gegenübertragung erschwert sei (3.11). Es beschrieben 6 % der Teilnehmenden (*n* = 9) die Nachteile, dass kein Settingwechsel, kein Abstand zum Alltag für die Patienten besteht sowie kein Wechsel zwischen Privatem und Beruflichen für die Therapeuten (3.12). Von den Teilnehmenden gaben 5 % (*n* = 7) an, dass der Rahmen weniger klar und das Setting künstlich sei (3.13). Eine weitere Kategorie ist, dass der Patient bzw. die Patientin ablenkbarer sei (3 %, *n* = 6, 3.14).

#### 4. Oberkategorie: Welche Interventionen, Therapietechniken sind bei VB nicht oder schlechter durchführbar?

Diese umfasst 8 Subkategorien. Von den Therapeuten benannten 6 % (*n* = 10), dass erlebniszentrierte Methoden, Imaginationen, nonverbale Methoden, Aufstellungen, Stuhlarbeit nicht möglich sind (4.1).

#### 5. Oberkategorie: Für wen ist VB nicht geeignet?

Diese umfasst 4 Subkategorien. Es schrieben 5 % (*n* = 8) der Therapeuten, dass VB für neue Patienten nicht geeignet ist (5.1), 4 % (*n* = 7), dass es schwierig bei Kindern, Familien und jüngeren Patienten sei (5.2). Es empfanden 3 % (*n* = 6), dass VB bei bestimmten Diagnosen nicht oder weniger geeignet ist (z. B. bei Patienten mit Persönlichkeits‑, Kommunikations‑, Angst‑, Zwangs‑, Bindungs‑, Körperbildstörungen und Selbstwertproblemen, 5.3).

#### 6. Oberkategorie: Wie positiv oder negativ erlebten Patienten und Therapeuten VB im Kontext der Pandemie?

Diese umfasst 12 Subkategorien. Die Hälfte der Therapeuten (50 %, *n* = 86) schrieb, dass VB gut angenommen wurde, auf Verständnis und Akzeptanz stieß, dass die Patienten aufgeschlossen, neugierig oder erfreut, erleichtert oder dankbar darüber waren, dass der Kontakt trotz Lockdown möglich war (6.1). Von den Teilnehmenden notierten 18 % (*n* = 31), dass Patienten VB ganz oder nach wenigen Sitzungen ablehnten oder die Therapie wegen der VB abbrachen (6.2). Es merkten 17 % (*n* = 29) an, dass VB keinen Einfluss auf den Therapieverlauf hatte (6.3); 14 % (*n* = 25) schrieben, dass VB zwar eine Notlösung und besser als nichts sei, jedoch nicht auf Dauer geeignet (6.4). Jeweils 8 % (*n* = 14 bzw. *n* = 13) erwähnten, dass die Patienten skeptisch (6.5) oder irritiert und verunsichert (6.6) waren. Als anstrengender für Therapeuten wurden VB von 7 % der Teilnehmenden (*n* = 12; 6.7) erlebt, 6 % (*n* = 10) waren nach anfänglicher Skepsis positiv überrascht (6.8) oder zeigten gemischte Reaktionen – teils Zustimmung, teils Ablehnung (6.9). Je 5 % (*n* = 9 bzw. *n* = 8) berichteten, dass sie nur wenige Stunden VB angeboten haben (6.10), bzw. dass die Patienten von VB oder technischen Problemen genervt waren (6.11). Weitere 7 Therapeuten (je 4 %) berichteten, dass die Patienten ungern wechselten, frustriert waren und die Präsenztherapie bevorzugen (6.12).

#### 7. Oberkategorie: Welche weiteren Beobachtungen in Bezug auf VB wurden beschrieben?

Diese umfasst 5 Subkategorien, die jeweils nur von einzelnen Therapeuten genannt wurden, und die sich nicht eindeutig den Vor- oder Nachteilen zuordnen ließen.

#### 8. Oberkategorie: Was half, VB umzusetzen?

Diese umfasst 14 Subkategorien. Hierzu gehören: kostenfreie gute Technik, gute Onlineprogramme (17 %, *n* = 29, 8.1), die Not und Notwendigkeit bzw. das Wissen, dass VB besser ist als keine Therapie (16 %, *n* = 28, 8.2), kollegialer Austausch (15 %, *n* = 26, 8.3), gute Unterstützung und Schulung durch KV und Ausbildungsinstitut (11 %, *n* = 20, 8.4), Flexibilität, Mut, Lust, Offenheit, Neugier (11 %, *n* = 19, 8.5), technisches Vorwissen sowie Vorerfahrungen mit VB (je 7 %, *n* = 12, 8.6, 8.7), dass der Wechsel auf VB nur stattfand, wenn Patienten dies wünschten, (5 %, *n* = 8, 8.8) oder die Offenheit und Akzeptanz der Patienten (4 %, *n* = 7, 8.9).

#### 9. Oberkategorie: Was erschwerte, VB umzusetzen?

Diese umfasst 3 Subkategorien. Hier wurden zum einen technische Probleme genannt, wie kein schnelles Internet, kein stabiles Netz, Bild blieb oft stehen, der Ton fragmentierte (46 %, *n* = 80, 9.1).

### Abhängigkeit der Nennungen vom Richtlinienverfahren

Im Folgenden wird narrativ beschrieben, wie sich die 4 Richtlinienverfahren im Hinblick auf die benannten Kategorien unterscheiden. Da der Vergleich für selten benannte Kategorien weniger zuverlässig ist, wird die Darstellung im Text auf Kategorien, die von *mehr als 25 teilnehmenden Therapeuten (14* *%) benannt* wurden, beschränkt. Weitere Details sind Abb. 1 zu entnehmen.

#### Generelle Vorteile der VB.

Dass VB räumliche Distanz überbrücken kann sowie örtliche und zeitliche Flexibilität ermöglicht, wurde von etwa jeweils der Hälfte der VT- und ST-Kollegen beschrieben, aber nur von maximal jedem dritten TP- oder jedem fünften AP-Kollegen. Dass VB vorteilhaft für Patienten ist, die nicht in die Praxis kommen können, wurde von ca. einem Fünftel der VT- und TP-Kollegen benannt, jedoch nur von 9–10 % der Vertreter der anderen Richtlinienverfahren.

#### Vorteile im Rahmen der Pandemie.

Dass die Kontinuität des therapeutischen Kontakts aufrechterhalten werden konnte, erwähnten 48 % bzw. 30 % der Psychodynamiker, aber nur rund 10–19 % der Vertreter der anderen Verfahren. Den Schutz vor dem Infektionsrisiko erwähnten ca. ein Drittel der VTler (35 %) und APler (29 %), im Vergleich zu 14–20 % der Vertreter anderer Verfahren.

#### Nachteile der VB.

Zwischen 25 und 33 % der VT-, TP- und AP-Kollegen beschrieben das Fehlen von Sinneseindrücken, Mimik, Gestik, Blickkontakt, Körpersprache, nonverbaler Kommunikation, aber nur 10 % der STler.

#### Wie erlebten Patienten und Therapeuten VB im Kontext der Pandemie?

Dass die Patienten aufgeschlossen, neugierig, erfreut, erleichtert oder dankbar darüber waren, dass der Kontakt trotz Lockdown möglich war, benannten in etwa zwei Dritteln der VT- und ST-Kollegen und die Hälfte der Psychodynamiker. Dass Patienten die OT ganz oder nach wenigen Sitzungen ablehnten oder die Therapie wegen der VB abbrachen, beschrieben in allen Verfahren 19–24 % der Teilnehmer, dass VB keinen Einfluss auf den Therapieverlauf hatte, 10 % (ST) bis ca. ein Viertel (VT) der Teilnehmer. Es erwähnten 29 % der APler sowie 13 % der VT-, 16 % der TP- und 20 % der ST-Kollegen, dass VB zwar als Notlösung geeignet und besser als nichts, jedoch nicht auf Dauer angelegt sei.

#### Was half, VB umzusetzen?

Die Hälfte der ST-Kollegen, aber nur 10 % bzw. 14 % der Psychodynamiker erwähnten kostenfreie gute Technik, gute Onlineprogramme. Die Not und Notwendigkeit bzw. das Wissen, dass VB besser ist als keine Therapie, benannten fast jeder fünfte TP-, AP- und VT- und etwa ein Drittel der ST-Kollegen, den kollegialen Austausch 10 % (ST) bis 20 % (TP) der Kollegen.

#### Was erschwerte, VB umzusetzen?

Es gaben 52 % TPler, 48 % APler, 40 % STler sowie ca. 59 % der VT-Kollegen technische Probleme an.

## Diskussion

### Ziel der Arbeit

Das Ziel dieser Studie war, möglichst differenziert – und daher mit qualitativer Methodik – darzustellen, welche Erfahrungen Psychotherapeuten mit der Durchführung von VB in Zeiten von COVID-19 gemacht haben. Ein Großteil der teilnehmenden Therapeuten hat die Möglichkeit genutzt, die für die qualitativen Analysen vorgesehenen Freifelder auszufüllen. Eine mögliche Interpretation dessen ist, dass es „Redebedarf“ gibt, dass also der plötzliche Anstieg von VB ein Thema ist, welches die Therapeuten beschäftigt.

### Erlebte Vor- und Nachteile von Videobehandlungen

Generell wurde ein breites, differenziertes Spektrum an Vor- und Nachteilen der VB beschrieben. Stellt man die am häufigsten genannten Vorteile (räumliche Distanz überbrücken, Kontinuität in der Behandlung aufrechtzuerhalten, örtliche und zeitliche Flexibilität, Zeit- und Kostenersparnis) den am häufigsten genannten Nachteilen gegenüber, fällt auf, dass es sich bei den Vorteilen um organisatorische Fakten oder pandemiebezogene Umstände handelt. Die Nachteile hingegen betreffen überwiegend die Interaktion zwischen Therapeut und Patient. Zu den hier am häufigsten beschriebenen Schwierigkeiten gehörten das Fehlen von Sinneseindrücken, Mimik, Gestik, Blickkontakt, nonverbaler Kommunikation, mitfühlendem Austausch, und ein vorhandenes Distanzgefühl. Diese Schwierigkeiten, die paralinguistische, körperliche und prosodische Aspekte der Kommunikation betreffen, wurden auch von anderen Autoren beschrieben (Fernández-Álvarez und Fernández-Álvarez 2021; DPtV 2020). In ihrem Bericht zur Arbeit mit Emotionen in videobasierter VB führten Thompson-de Benoit und Kramer (2020) aus, dass diese Arbeit aus der Ferne zwar prinzipiell machbar sei, jedoch spezifische Herausforderungen beim Vertiefen von Emotionen und beim Umgang mit dysregulierten emotionalen Erfahrungen entstehen, durch das Fehlen von direktem Blickkontakt, die erschwerte Nutzung des Tonfalls oder die Körperhaltung sowie schwierigere Einschwingungs- und Abstimmungsprozesse. Auch die von der DPtV durchgeführte und veröffentlichte „Blitzumfrage“ zur VB kommt zu dem Ergebnis, dass die nonverbale Interaktion, die Nutzung von Sinneseindrücken, diagnostische und Beziehungsarbeit via Video erschwert sind (DPtV 2020).

An dieser Stelle muss jedoch auch ergänzt werden, dass in einer Reihe von Studien gezeigt wurde, dass eine gute therapeutische Allianz im Rahmen von VB hergestellt werden kann und oft zur Face-to-Face-Therapie vergleichbare Werte in Allianz-Ratings erreicht werden können (Simpson und Reid 2014; Cavanagh und Millings 2013; Sucala et al. 2012). Interessanterweise zeigen sich Diskrepanzen in der Allianzeinschätzung im Vergleich von VB und Face-to-Face-Therapien am ehesten aus Sicht der Therapeuten, nicht jedoch aus der Perspektive der Patienten. So deuten die Ergebnisse einiger Studien auch darauf hin, dass die therapeuteneingeschätzte Allianz im Rahmen der VB niedriger ist, als dies in Face-to-Face-Therapien der Fall ist (Cataldo et al. 2021; Rees und Stone 2005). Die Ursachen für diese z. T. widersprüchlichen Erfahrungen von Patienten und Therapeuten näher zu untersuchen, bleibt weiter von Bedeutung. Die in der aktuellen Untersuchung von den Therapeuten benannten Einschränkungen und Nachteile bieten wichtige Anhaltspunkte. Möglicherweise werden mit den gängigen Instrumenten zur Erfassung der Allianz auch die körperlichen und prosodischen Aspekte der Interaktion weniger sensitiv erfasst, oder das Fehlen dieser Aspekte bildet sich in den niedrigeren Werten der Therapeuten ab.

### Verfahrensspezifische Besonderheiten und verfahrensübergreifende Einschränkungen

Bisherige Studien geben auch Hinweise auf Unterschiede zwischen den therapeutischen Verfahren bei der Bewertung der VB. So fand eine Untersuchung unter schwedischen und deutschen Psychotherapeuten, dass kognitive Verhaltenstherapeuten die VB positiver einschätzten als psychodynamische Kollegen (Schuster et al. 2020). Dieser Befund deckt sich mit den vorgestellten aktuellen Ergebnissen. Im Vergleich der Richtlinienverfahren fiel in der vorliegenden Studie auf, dass VT-Kollegen eher allgemeine Vorteile wie das Überbrücken von räumlicher Distanz oder zeitliche Flexibilität benannten, AP-Kollegen dagegen stärker die Kontinuität der Beziehung in Pandemiezeiten betonten. Passend dazu bewerteten die AP-Kollegen VB häufiger als „gute Notlösung“, die aber nicht auf Dauer geeignet ist, wohingegen die VTler und STler häufiger als die Psychodynamiker angaben, dass ihre Patienten gute Akzeptanz, Verständnis, Dankbarkeit als Reaktion auf die VB zeigten, jedoch einige Interventionen (beispielsweise Stuhlarbeit, Exposition, Imagination) nur schwer möglich seien.

Verfahrensübergreifend wurde berichtet, dass VB nicht für alle Patienten und Störungsbilder geeignet sei, was im Einklang mit den Ergebnissen der eingangs erwähnten niederländischen Studie steht (Feijt et al. 2020). Einige Therapeuten erwähnten explizit, dass das Format für neue Patienten und bei Kindern oder jüngeren Patienten ungeeignet sei. Dies deckt sich mit den Ergebnissen der Umfrage der DPtV (2020). Einige Therapeuten erwähnten auch, dass es Patienten gab, die von der Distanz profitierten.

Fast die Hälfte der Therapeuten, die an der Erhebung teilgenommen haben, beschrieb, dass technische Probleme die Durchführung der VB erschwerten. Dieses Problem, dass Zeit zur Lösung technischer Probleme statt für das Engagement für die Therapie verwendet wird, wurde auch von anderen Autoren aufgegriffen (Markowitz et al. 2021). Als erleichternde Bedingungen in der Erhebung wurden der Gedanke an die pure Notwendigkeit (besser VB als keine Therapie), gute Technik sowie der kollegiale Austausch genannt. Das weist darauf hin, dass viele Therapeuten von einer Plattform für Schulungen und Austausch profitieren könnten, beispielsweise integriert ins Curriculum der Psychotherapeutenausbildung, im Rahmen von Weiterbildungen, über die Kammern, Berufsverbände oder Institute.

Prinzipiell ist festzuhalten, dass Psychotherapeuten der Möglichkeit, Videobehandlungen durchzuführen, mehrheitlich aufgeschlossen gegenüberstehen. Gleichzeitig zieht die Mehrheit der Therapeuten allerdings eine Behandlung im persönlichen Kontakt der Videobehandlung vor und schätzt Sitzungen im persönlichen Kontakt wirksamer ein als Videositzungen (DPtV 2020; Beck-Hiestermann et al. 2021). Im Hinblick auf die Akzeptanz unter den Patienten berichtete fast die Hälfte der Therapeuten in vorliegender Studie, dass die Patienten das Format gut angenommen hätten. Mehr als ein Sechstel berichtete auch davon, dass Patienten OT ablehnten oder die Therapie aufgrund des Formats abbrachen. Ein knappes Siebtel notierte, dass VB nur als befristete Notlösung geeignet sei, und dass Patienten ungern wechselten, frustriert waren und Präsenztherapie bevorzugen. Die im April 2020 durchgeführte Umfrage der DPtV, an der 4466 in der ambulanten Versorgung tätige Therapeuten teilnahmen, ergab, dass 20 % der Patienten es ablehnten, Psychotherapie per Video durchzuführen, und dass mehr als die Hälfte der Patienten weiterhin im persönlichen Kontakt behandelt wurden (DPtV 2020).

### Implikationen für weiterführende Forschungen

Erfahrungen von Klinikern sind ein wertvoller Ausgangspunkt für weiterführende Forschungen zu den Chancen und Risiken der VB für die psychotherapeutische Versorgung unterschiedlicher Patientengruppen. Angesichts der großen Relevanz der Überzeugtheit der Therapeuten vom angewandten Verfahren (Allegiance; Wampold et al. 2018) ist die Perspektive der Versorger zum einen essenziell. Zum anderen liefern die Aussagen der Kliniker, v. a. jene, die über verschiedene Studien hinweg übereinstimmen, bedeutsame Hypothesen für weiterführende Forschungen. So ist es aus Sicht der Autoren ein zentrales Anliegen, die Bedeutung paralinguistischer, körperlicher und prosodischer Aspekte im therapeutischen Dialog und diesbezügliche Unterschiede zwischen Face-to-face-Therapien und VB weiterführend zu erforschen.

### Limitationen

Die vorliegende qualitative Analyse basiert auf einer großen Stichprobe, die alle Richtlinienverfahren einbezog. Die onlinebasierte Erhebung trug zur schnellen Rekrutierung vieler Teilnehmer bei, könnte jedoch auch einen Selektionsbias hin zu technikaffinen Studienteilnehmern mit sich gebracht haben, der bei der Bewertung der Ergebnisse berücksichtigt werden muss. Beim Vergleich der Charakteristika der aktuell rekrutierten Stichprobe mit der Gesamtheit der an der kassenärztlichen Versorgung teilnehmenden Psychotherapeuten erscheint die prozentuale Verteilung der Richtlinienverfahren in der vorliegenden Studie relativ repräsentativ (mit einem Vorherrschen der VT, gefolgt von der TP). Die Teilnehmer der aktuellen Studie waren im Durchschnitt jedoch deutlich jünger. Dies liegt höchstwahrscheinlich im Einbezug von Ausbildungsteilnehmern begründet. Das jüngere Durchschnittsalter könnte allerdings ebenso wie der Erhebungsmodus zu einer Verzerrung hin zu Teilnehmern mit einer größeren Affinität zu moderner Technik geführt haben. Zudem sollte das qualitativ entwickelte Kategoriensystem perspektivisch quantitativ überprüft werden.

## Schlussfolgerung und Ausblick

Längerfristig stellt sich die Frage, welche Rolle VB auch nach Abklingen der Pandemie in der Behandlungslandschaft einnehmen wird. Für viele Patienten und Therapeuten wird sie vermutlich durch die erlebten Einschränkungen in der therapeutischen Beziehungsgestaltung und durch mangelnde Umsetzbarkeit von Interventionen eine Notlösung bleiben. Gleichzeitig ist es durchaus vorstellbar, dass sie für einige Patienten mehr als eine „Überbrückung“ ist. Insbesondere bei bekannten Problemen wie therapeutischer Unterversorgung auf dem Land oder vulnerablen Gruppen, die nicht in die Praxis kommen können (Mütter im Wochenbett, schwer kranke oder mobilitätseingeschränkte Patienten) kann VB einen Beitrag zur flächendeckenden Versorgung leisten. Welche Rolle das Fehlen paralinguistischer, körperlicher und prosodischer Aspekte im therapeutischen Dialog im Hinblick auf die Qualität der Psychotherapie spielt, sollte weiterführend erforscht werden.

## Fazit für die Praxis

- Während des ersten Lockdowns (März bis Mai 2020) boten Therapeuten vermehrt Videobehandlungen an (VB; Anstieg von 20 % auf 43 %).
- Fast die Hälfte der Therapeuten beschrieb, dass die Patienten das Format gut angenommen hätten. Die Therapeuten benannten ein differenziertes Spektrum an Vor- und Nachteilen der VB.
- Besonders häufig genannte Vorteile waren die örtliche und zeitliche Flexibilität, die Kontinuität des Kontaktes in Pandemiezeiten und die Vermeidung des Infektionsrisikos.
- Die meistbenannten Nachteile waren, dass Sinneseindrücke, Mimik, Gestik, Blickkontakt und Körpersprache fehlten, dass ein Distanzgefühl entstand, dass weniger Bezogenheit, Atmosphäre und mitfühlender Austausch spürbar waren, und dass Prozesse des Sich-aufeinander-Einschwingens fehlten.
- Es gibt verfahrensspezifische Unterschiede bei der Bewertung der VB. Verhaltenstherapeuten benannten häufiger positive Aspekte der VB als die psychodynamischen Kollegen und wiesen eher auf allgemeine Vorteile wie das Überbrücken von räumlicher Distanz oder zeitliche Flexibilität hin. Kollegen aus der analytischen Psychotherapie (AP) dagegen betonten stärker die Kontinuität der Beziehung in Pandemiezeiten.
- Technische Probleme erschwerten die Umsetzung.
- Viele Therapeuten empfanden VB als „Notlösung“, die nicht auf Dauer angelegt sei. Allerdings könnten VB über die Pandemiezeit hinaus helfen, bekannte Versorgungsprobleme zu lösen.
